# Arginine Depletion by Arginine Deiminase Does Not Affect Whole Protein Metabolism or Muscle Fractional Protein Synthesis Rate in Mice

**DOI:** 10.1371/journal.pone.0119801

**Published:** 2015-03-16

**Authors:** Juan C. Marini, Inka Cajo Didelija

**Affiliations:** 1 Section of Critical Care Medicine, Department of Pediatrics, Baylor College of Medicine, Houston, Texas, United States of America; 2 United States Department of Agriculture/Agricultural Research Service Children’s Nutrition Research Center, Department of Pediatrics, Baylor College of Medicine, Houston, Texas, United States of America; Monash University, AUSTRALIA

## Abstract

Due to the absolute need for arginine that certain cancer cells have, arginine depletion is a therapy in clinical trials to treat several types of cancers. Arginine is an amino acids utilized not only as a precursor for other important molecules, but also for protein synthesis. Because arginine depletion can potentially exacerbate the progressive loss of body weight, and especially lean body mass, in cancer patients we determined the effect of arginine depletion by pegylated arginine deiminase (ADI-PEG 20) on whole body protein synthesis and fractional protein synthesis rate in multiple tissues of mice. ADI-PEG 20 successfully depleted circulating arginine (<1 μmol/L), and increased citrulline concentration more than tenfold. Body weight and body composition, however, were not affected by ADI-PEG 20. Despite the depletion of arginine, whole body protein synthesis and breakdown were maintained in the ADI-PEG 20 treated mice. The fractional protein synthesis rate of muscle was also not affected by arginine depletion. Most tissues (liver, kidney, spleen, heart, lungs, stomach, small and large intestine, pancreas) were able to maintain their fractional protein synthesis rate; however, the fractional protein synthesis rate of brain, thymus and testicles was reduced due to the ADI-PEG 20 treatment. Furthermore, these results were confirmed by the incorporation of ureido [^14^C]citrulline, which indicate the local conversion into arginine, into protein. In conclusion, the intracellular recycling pathway of citrulline is able to provide enough arginine to maintain protein synthesis rate and prevent the loss of lean body mass and body weight.

## Introduction

Cachexia is a multifactorial syndrome characterized by a progressive loss of body weight [1] and especially of lean body mass [2]. Decreased protein synthesis and increased protein degradation have been described in cancer patients and animal models [3,4,5]. Although different protein compartments may react differently to the presence of the tumor, skeletal muscle appears to be one of the most compromised, with a reduction in size [4,5] and function [6,7]. This is very relevant in cancer patients because loss of muscle and lean body mass results in a worse prognosis, even after accounting for other factors such as age [8].

In many species, including humans, arginine is considered a semi-essential amino acid because under certain conditions endogenous synthesis cannot meet the demand for this amino acid [9]. Arginine is not only a building block required for protein synthesis, but it also has a direct effect on mTOR and the translation initiation pathway [10]. In addition, arginine is the only precursor for nitric oxide synthesis. Because this signaling molecule is a key component in the regulation of blood flow and other metabolic processes, arginine may regulate the supply of nutrients and their utilization in muscle and other tissues [11]. The endogenous synthesis of arginine is a multi-organ process that involves the synthesis of citrulline by the gut and further conversion into arginine by the kidney [12], where argininosuccinate synthase and lyase (ASS and ASL, enzymes that catalyze the conversion of citrulline into arginine) are highly expressed. In addition, these two enzymes are expressed in multiple tissues and it is likely that a fraction of the citrulline produced in the gut is utilized directly by the tissues to meet, at least partially, their arginine needs [13,14].

Certain cancer types lack ASS or ASL, and thus these cancer cells are auxotrophic for arginine and depend on the provision of this amino acid from the blood to sustain protein synthesis and growth [15,16]. Arginine deiminase is a bacterial enzyme that catalyzes the hydrolysis of the imino group of arginine, producing citrulline and ammonia [17]. When this enzyme is given to mammals it causes a depletion of circulating arginine [18]. Pegylated arginine deiminase (ADI-PEG 20) has been formulated to increase the half-life (~6 d) of the enzyme and has been used in clinical trials with some success in cancer patients [19,20,21]. Although no negative effect of ADI-PEG 20 on body weight of cancer patients has been reported, the effects of arginine depletion on whole body and tissue protein synthesis have not been studied. Given the importance of lean mass and body weight maintenance in the survival and prognosis of cancer patients this is an area that warrants further investigation.

The objective of the present research was to determine the effect of arginine depletion by ADI-PEG 20 on whole body protein synthesis and fractional protein synthesis rate in multiple tissues of mice.

## Materials and Methods

### Animals and housing

Six week old male (30–35 g BW) Institute of Cancer Research (ICR) mice (Hsd:ICR (CD-1)Harlan Laboratories, Houston, TX) were used in all the experiments described below. Mice were housed in a SPF facility (4 per cage) and fed an irradiated 18% crude protein feed (Harlan Teklad, Rodent Diet 2920X) and autoclaved reverse osmosis water was available at all times. Mice were under a 12 h light cycle (0600 to 1800 h) in a temperature (22±2°C) and humidity (55±5%) controlled environment. This study was carried out in strict accordance with the recommendations in the Guide for the Care and Use of Laboratory Animals of the National Institutes of Health. The protocol was approved by Baylor College of Medicine Institutional Animal Care and Use Committee (Animal Protocol AN-4496).

For all the studies described below, mice either received 5 IU ADI-PEG 20 (Polaris Pharmaceutical, Inc., San Diego, CA) intramuscularly or the same volume (50 μL) of saline via the same route. Mice were studied 6 days after drug administration. Body weight and composition (QMR, EchoMRI-100, EchoMRI LLC, Houston, TX) was determined before and 6 days after treatment administration.

### Whole body protein kinetics

Six days after ADI-PEG 20 treatment, feed was removed at 7:00 and mice (n = 8) weighed at 9:30. After a 3 h feed deprivation, mice were restrained and a tail vein catheter was inserted as previously described [22,23]. The tail vein catheter was then connected to syringe infusion pumps (PHD2000, Harvard Apparatus Inc., Holliston, MA) for the 4 h infusion.

We have previously shown that with this the prime-continuous infusion protocol amino acid and their products reached plateau enrichment which allows for the utilization of steady state kinetic models [22]. A primed-continuous infusion of (ring) [^2^H_5_]phenylalanine (16 μmol•kg^-1^; 16 μmol•kg^-1^•h^-1^) and [3,5–^2^H_2_]tyrosine (10 μmol•kg^-1^; 10 μmol•kg^-1^•h^-1^) were used to determine phenylalanine and tyrosine fluxes and phenylalanine hydroxylation rate. After the 4 h infusion, blood was drawn from the submaxibular bundle, centrifuged and plasma stored at -80°C.

### Tissue specific protein synthesis

After a 3 h feed deprivation, mice (n = 10) received a flooding dose of (ring) [^2^H_5_]phenylalanine (150 mmol/L, 10 mL/kg BW) using a tail vein catheter. After 12 min mice were decapitated and trunk blood was collected. Blood was centrifuged at *1500 x g* for 15 min at 4°C and plasma was kept frozen at -80°C. Tissues (liver, kidney, spleen, heart, lungs, stomach, small and large intestine, pancreas, gastrocnemious muscle, brain, testis and thymus) were collected immediately and snap frozen into liquid nitrogen. Tissues were kept at -80°C until analysis.

### Citrulline incorporation into protein

After a 3 h feed deprivation, mice (n = 5) received a bolus dose of [^14^C] citrulline (110 KBq) using a tail vein catheter. Mice were decapitated 1 h later and blood and tissues collected as described before.

### Analysis

Arginine and citrulline plasma concentrations were determined by LC-MS/MS as their dansyl derivatives as described elsewhere [24]. Plasma phenylalanine and tyrosine enrichments were also determined by LC-MS/MS as dansyl derivatives. Tissue was homogenized and protein precipitated with perchloric acid (PCA) to determine tissue specific protein synthesis, and the incorporation of [^14^C] into protein. After thoroughly washing the protein pellet to remove any traces of soluble intra- and extracellular contaminants, including free tracer, the pellet was solubilized in HCl and hydrolyzed for 24 h at 110°C to determine the incorporation of [^2^H_5_]phenylalanine into protein. After drying, the resulting amino acids were derivatized with dansyl chloride as indicated earlier and enrichments determined by LC-MS/MS. To determine the incorporation of [^14^C] into protein, the protein pellet was solubilized with Soluene-350 (PerkinElmer, Waltham MA) at 60°C for 4 h. Then scintillation cocktail Ultima Gold (PerkinElmer) was added and ^14^C radioactivity was measured using a Liquid Scintillation Analyzer (Packard TriCarb 3180 TR). Tissue of similar animals under identical conditions, but not infused with [^14^C] citrulline was used for background correction.

### Calculations

To determine whole body protein turnover a phenylalanine-tyrosine kinetic model was utilized [25]. In this model, during fasting, the rate of appearance of phenylalanine represents protein breakdown and the irreversible loss of phenylalanine is given by hydroxylation (conversion into tyrosine). The phenylalanine that is not lost is assumed to be reutilized and represent the rate of whole body protein synthesis.

The rates of appearance of phenylalanine and tyrosine were calculated from the isotopic dilution of the intravenously infused tracers at plateau enrichment, as described by the equation:

$$Ra_{Aa}=i_{Aa}•(\frac{E_{iAa}}{E_{Aa}}-1)$$ Eq. [1]

where *Ra*_*Aa*_ is the rate of appearance (flux) of the unlabeled amino acid *Aa* (μmol•kg^-1^•h^-1^), *i*_*Aa*_ is the intravenous infusion rate (μmol•kg^-1^•h^-1^), *E*_*iAa*_ is the enrichment of the infused amino acid tracer and *E*_*Aa*_ is the plasma enrichment of the amino acid *Aa* at isotopic plateau enrichment (mpe).

The rate of interconversion of phenylalanine to tyrosine (hydroxylation rate), was calculated based on the transfer of the label from the precursor to the product as

$$Rc_{prec\to prod}=Ra_{prod}•\left(E_{prod}/(100-E_{prec})\right)/\left(E_{prec}/(100-E_{prec})\right)$$ Eq. [2]

where *Rc*_*prec→prod*_ is the rate of conversion of the precursor into the product (μmol•kg^-1^•h^-1^), *Ra*_*prod*_ is the rate of appearance of the product, *E*_*prod*_ and *E*_*prec*_ are the plasma enrichments of the of the product and precursor, respectively.

Protein synthesis rate was determined by the incorporation of the phenylalanine tracer in protein as

$$FSR=\left(E_{tissue}/E_{phe}\right)•\left(1440/12\right)•100$$ Eq. [3]

where (FSR) is fractional protein synthesis rate (%/d), *E*_*tissue*_ is the enrichment of phenylalanine in tissue, *E*_*phe*_ *is* the enrichment of plasma phenylalanine, 1440 the number of minutes in a day and 12 the time in minutes between the phenylalanine bolus dose and the tissue collection.

We used a complementary approach to protein FSR to determine the tissue utilization of citrulline for arginine synthesis when the interorgan supply of arginine is impaired by ADI-PEG 20. To determine this local process we measured the incorporation of the ureido [^14^C] of citrulline into the acid-precipitable fraction of different tissues. This approach assumes that: (1) there is no tRNA for citrulline and thus no (direct) incorporation of citrulline into protein; (2) the new arginine that is formed from citrulline can then be incorporated into protein and, in ADI-PEG 20 treated mice, this process reflects a local phenomenon (note that if this arginine leaves the cell will be immediately converted in to citrulline by the circulating ADI-PEG 20); (3) protein is the main component of the acid-precipitable fraction and; (4) the main fate of the ureido carbon of citrulline is urea which is lost from the system, i.e., the ureido carbon does not recycle (this is also true for other minor pathways, e.g. agmatine and creatine synthesis).

### Data analysis

Data were analyzed statistically as a complete randomized design utilizing the proc mixed procedure of SAS (v. 9.2, SAS Inst, Inc., Cary, NC). Means were tested for significance at the 5% level.

## Results

Mice gained weight during the 6 days after treatment (0.31±0.04 g/d). However, no differences (P > 0.15) in body weight (Control: 33.9±0.64 g; ADI-PEG 20: 33.6±0.64 g) or body composition (Control: lean 25.0±0.67 g; fat 4.7±0.36 g; ADI-PEG 20: lean 25.2±0.48 g; fat 4.5±0.34 g) were observed between the ADI-PEG 20 and the Control groups. Plasma arginine in Control mice was 213±32 μmol/L, but it was barely detectable (< 1 μmol/L) in ADI-PEG 20 treated mice (P<0.001). In contrast, plasma citrulline concentration was more than tenfold greater (P<0.001) in the mice treated with ADI-PEG 20 when compared with the control animals (962±115 μmol/L vs 82±4 μmol/L, respectively).Plasma ornithine concentration was reduced (P<0.001) in mice that received the ADI-PEG 20 treatment (67±3 μmol/L vs 112±11 μmol/L).

**Fig 1:**
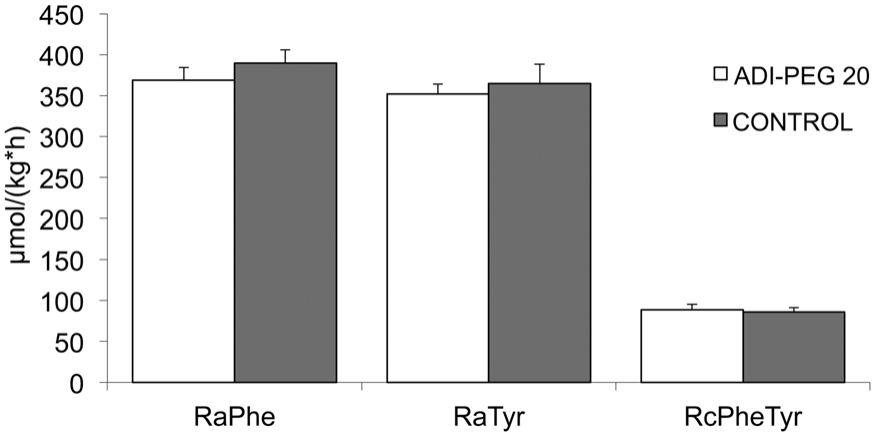
Rate of appearance (Ra) of phenylalanine and tyrosine, and rate of conversion of phenylalanine into tyrosine (Rc; hydroxylation) in mice treated with 5 IU ADI-PEG 20 or saline (Control). Bars are means±SE, n = 8.

**Fig 2:**
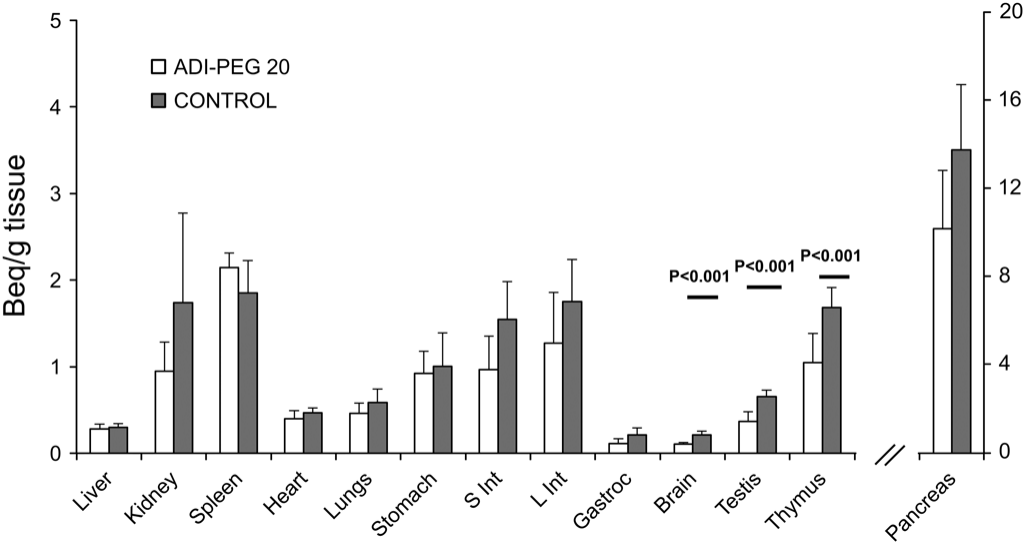
[^14^C] citrulline incorporation into protein of multiple tissues of mice treated with 5 IU ADI-PEG 20 or saline (Control). Bars are means±SE, n = 5.

There was no effect of ADI-PEG 20 on the rates of appearance of phenylalanine (P = 0.19) and tyrosine (P = 0.32; Fig. 1). Likewise, there was no difference in the rate of conversion of phenylalanine into tyrosine (P = 0.39; Fig. 1.). ADI-PEG 20 had no effect on protein FSR of most tissues; however, protein FSR of testis, thymus and brain was reduced (P < 0.02) due to ADI-PEG 20 treatment (Table 1). A wide range of ^14^C citrulline incorporation was observed in tissues of Control and ADI-PEG 20 treated mice (Fig. 2), but a reduction (P < 0.01) was only detected for testis, thymus and brain of mice in the ADI-PEG 20 treated group.

**Table 1:** Fractional protein synthesis rate of tissues in mice treated with 5 IU ADI-PEG 20 or saline (Control).

Tissue	ADI-PEG 20	Control	P <
	%/d	%/d	
Pancreas	443.5±21.3^a^	426.6±26.2	0.623
Stomach	109.0±4.0	107.5±5.3	0.824
Liver	107.0±5.9	103.2±4.1	0.604
Large Intestine	84.8±4.5	78.3±7.3	0.457
Small Intestine	80.0±4.7	78.3±3.5	0.775
Spleen	73.3±4.1	61.9±3.7	0.069
Kidney	69.6±4.3	65.4±1.9	0.378
Thymus	48.6±2.9	58.7±2.3	0.015
Testis	33.3±1.1	36.8±0.6	0.014
Lung	18.7±1.7	18.2±1.7	0.840
Heart	17.0±0.4	15.9±0.4	0.077
Muscle	11.3±0.7	10.0±0.4	0.122
Brain	8.6±0.3	9.8±0.4	0.024

^a^Values are means±SE, n = 10

## Discussion

Up to 50% of cancer patients suffer from muscle atrophy and body protein loss [26]. Furthermore, the extent of body weight loss correlates with shorter survival [27] and for this reason multiple strategies have been proposed to reduce the rate of weight and protein loss in cancer patients [28,29,30,31].

The maintenance of cell function and body protein stores requires the availability of all 20 proteogenic amino acids to sustain protein synthesis. If the availability of any amino acid is reduced and becomes limiting, protein synthesis is impaired. Arginine is considered a semi-essential amino acid because under certain conditions the endogenous synthesis cannot meet its metabolic demand [9]. However, due to the widespread presence of ASS and ASL [32,33] it is not arginine itself what is limiting but its precursor, citrulline. Because certain cancer types lack ASS or ASL, these cancer cells are auxotrophic for arginine and rely on the provision of this amino acid from the blood to sustain protein synthesis and growth [16,34].

As previously shown by others [18], ADI-PEG 20 caused the depletion of the circulating arginine pool, but increased the plasma concentration of citrulline. ADI-PEG 20 had no effect on body weight gain or body composition. As expected, the rates of appearance of phenylalanine and its conversion into tyrosine were not affected by ADI-PEG 20 administration. This indicates that whole body protein synthesis and degradation are not affected when arginine is depleted by ADI-PEG 20. Arginine depletion with arginase, however, results in severe weight loss and death [35], that can be rescued with citrulline supplementation. This demonstrate that while ADI-PEG 20 causes a depletion of circulating arginine, this depletion is not systemic due to the ability of multiple tissues to derive arginine from citrulline.

Further indication of systemic arginine sufficiency was that ADI-PEG 20 treated mice showed no differences in the fractional protein synthesis rate of most tissues (including skeletal muscle). However, brain, testis and thymus of treated mice showed a reduction in protein synthesis, which seems to indicate that these tissues were not able to synthesize enough arginine to sustain protein synthesis. Interestingly, old reports in rats showed that homogenates of testis and brain (thymus was not determined) [36] were unable to produce arginine from citrulline. The incorporation of [^14^C] from citrulline into tissue protein in the ADI-PEG 20 treated mice, however, demonstrates that these organs do produce arginine, which is consistent with the presence of ASS and ASL in these tissues [32,33]. These results, however, seem to contrast with the expression of ASL and ASS reported in mice [32]. For example, whereas a modest ASL and ASS expression (and of similar magnitude) was reported for testes and pancreas, the activity we detected in vivo were very different. This exemplifies one of the limitations of inferring in vivo function from expression data.

In conclusion, arginine depletion with ADI-PEG 20 did not affect whole body protein metabolism nor protein FSR of most tissues. Thus concerns that arginine depletion may negatively affect protein synthesis and exacerbate the cachexia observed in cancer seem not to be warranted. The high citrulline concentration that results from the deimination of arginine is able to provide enough precursor for the synthesis of intracellular arginine and sustain systemic arginine availability. This is an advantage of arginine depletion by ADI-PEG 20 over arginase, which produces ornithine that cannot be utilized for the re-synthesis of the arginine molecule, and relies in the provision of exogenous citrulline to prevent body weight loss and death.
